# Tuning the Mechanical Properties of Crosslinked Copolymers via Sequence and Solvent‐Selective Swelling for Vat Photopolymerization

**DOI:** 10.1002/anie.8144140

**Published:** 2026-06-24

**Authors:** Chia‐Min Hsieh, Krista G. Schoonover, Naushad Ahmed, Jung‐Bin Ahn, Shuo Qian, Michael J. A. Hore, Weiling Xia, Kaiwen Hsiao, Yue Yuan, Mani Sengoden, Donald J. Darensbourg, Emily Pentzer, Peiran Wei

**Affiliations:** ^1^ Department of Chemistry Texas A&M University College Station Texas USA; ^2^ Department of Materials Science and Engineering Soft Matter Facility Texas A&M University College Station Texas USA; ^3^ Neutron Scattering Division and Second Target Station Oak Ridge National Laboratory Oak Ridge Tennessee USA; ^4^ Department of Macromolecular Science and Engineering Case Western Reserve University Cleveland Ohio USA; ^5^ Department of Materials Science and Engineering Texas A&M University College Station Texas USA; ^6^ Center for Nanophase Materials Sciences Oak Ridge National Laboratory Oak Ridge Tennessee USA

**Keywords:** additive manufacturing, block copolymer, mechanical properties, microphase separation, polymer architecture, solvation effect

## Abstract

Block copolymers (BCPs) offer distinct advantages for vat photopolymerization by enabling mechanically programmable network structures through microphase‐separated morphologies that can be kinetically trapped during curing, yielding properties unattainable in homogeneous resins. However, the respective roles of repeat‐unit sequence and solvent environment, together with their interplay in directing network formation and mechanical performance, remain unclear. Here, we synthesize a series of CO_2_‐based polycarbonate copolymers comprising a crosslinkable glassy poly(vinyl cyclohexene carbonate) (PVCHC, A block) and a non‐crosslinkable soft poly(propylene carbonate) (PPC, B block). The polymer sequence is systematically varied (ABA, BAB, and statistical), and solvent choice controls block‐selective swelling to jointly control gelation behavior, microphase morphology, and mechanical response through changes in the accessibility and local environment of photocrosslinkable vinyl groups during network formation, as revealed by photorheology and small angle x‐ray scattering. By tuning polymer sequence and curing solvent, we transform nominally identical formulations from brittle to highly ductile materials, achieving a three‐orders‐of‐magnitude range in toughness (0.003 to 9.1 MJ m^−3^). These results establish clear structure–processing–property relationships and identify polymer sequence and selective solvation as powerful strategies for programming both printability and performance of block copolymer resins for additive manufacturing.

## Graphical abstract

Polymer sequence and solvent selectivity independently modulate pendant‐vinyl accessibility during photocuring, trapping different topologies in CLIP and controlling mechanical properties.

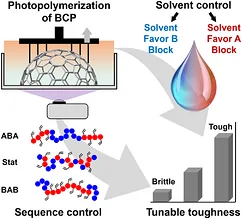

## 1 Introduction

Block copolymers (BCPs) represent a unique class of macromolecular materials whose physicochemical properties differ markedly from those of homopolymers, blends, and statistical copolymers [1, 2]. This uniqueness stems from their ability to undergo microphase separation, in which chemically distinct polymer blocks segregate into ordered nanostructures while remaining covalently tethered [3, 4]. The resulting morphologies, such as spheres, cylinders, gyroids, and lamellae, typically exhibit characteristic length scales of 10–100 nm [5, 6]. These nanostructures can be realized through tailoring molar mass, composition, and block architecture, giving control over mechanical, optical, and thermal properties [6, 7]. Commercial products, such as Kraton styrenic BCPs (e.g., SBS thermoplastic elastomers) and Pluronic PEO–PPO–PEO triblocks, highlight the broad impact of BCPs in adhesives, elastomers, surfactants, and drug‐delivery formulations [8, 9, 10].

Despite their distinctive properties and commercial success, most BCP applications have traditionally relied on conventional processing routes (e.g., extrusion, molding, solution casting) [11], which constrain macroscopic geometric complexity and limit the integration of structural features across multiple length scales within a single design [12]. The advent of additive manufacturing (3D printing) has opened new opportunities for BCPs by enabling fabrication of architected structures with simultaneous control over macroscopic geometry and microscopic morphology [13, 14, 15, 16]. For example, in extrusion‐based printing, bottlebrush BCP inks have been used in direct ink writing to produce bottlebrush BCP photonic crystals, where varying deposition conditions during printing kinetically trap different lamellar d‐spacings, thereby shifting the Bragg reflection and generating structural color across the visible spectrum [17]. In a separate approach, BCP self‐assemblies have been employed to generate micro‐organogel supports for precision embedded printing of silicone [18]. In parallel, vat photopolymerization (VPP) techniques, including stereolithography, digital light processing (DLP), and two‐photon polymerization (2PP), have also proven effective for printing BCP‐based materials, for instance, to realize pH‐responsive soft actuators [19], thermo‐responsive shape‐morphing materials [20, 21, 22], and elastomeric thermosets [23].

Unlike extrusion‐based printing, VPP forms covalent networks in situ; thus, curing not only defines the macroscopic part geometry but can also immobilize the underlying microscopic morphology. In BCP‐based resins, this kinetic trapping of microphase separation enables high‐resolution 3D printing of parts that retain distinct local nanostructures [19, 20, 23, 24, 25]. Recent work by Blasco and co‐workers exemplifies this potential: pre‐self‐assembled PS‐*b*‐PMMA‐based inks were used in 2PP printing to generate complex 3D microstructures with lamellar or cylindrical order preserved through the entire object [13]. The ability of BCP‐based resins to carry self‐assembled morphologies into printed structures provides powerful handles to tailor bulk properties, but to date most studies treat BCP molar mass, block ratio, and overall crosslink density as the primary design variables. The roles of block sequence, solvent choice, and their interplay in directing network formation, trapped morphology, and mechanical performance remain poorly understood, limiting the rational design of BCP‐based resins for precision mechanical targets.

**FIGURE 1:**
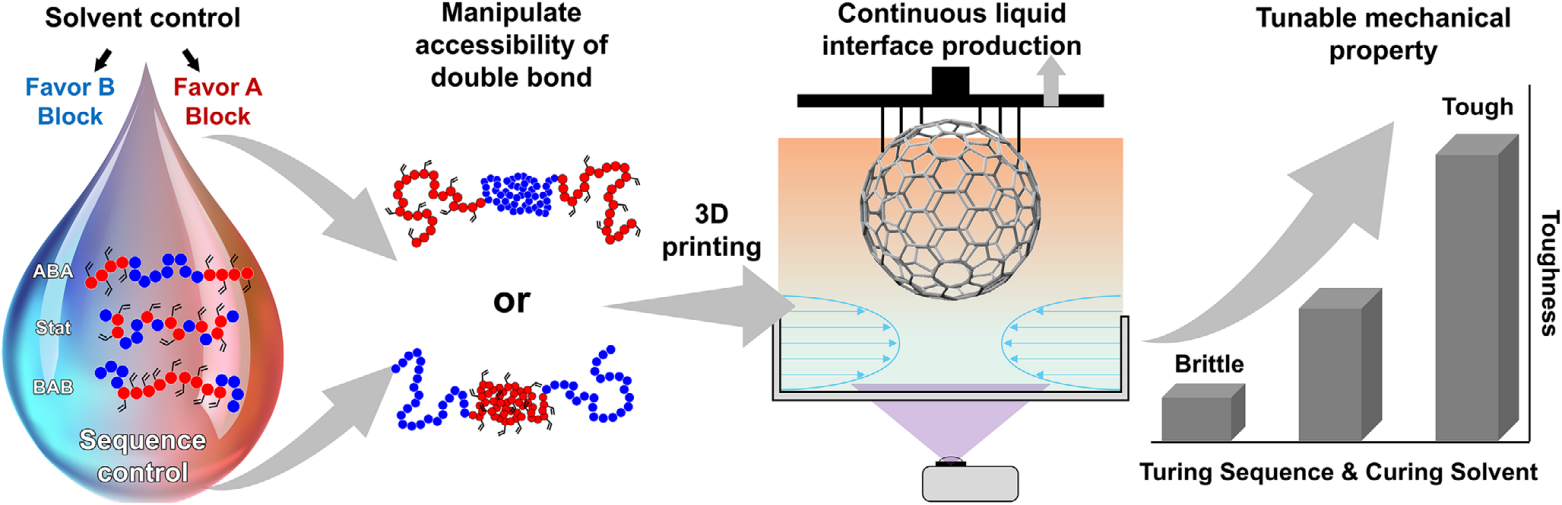
Polymer sequence (ABA/BAB/statistical) and solvent selectivity modulate pendant‐vinyl accessibility during photocuring, trapping different topologies in CLIP and controlling mechanical response (tough vs. brittle).

In this work, we address this gap by combining sequence control with solvent‐selective swelling as orthogonal knobs to program crosslinked BCP networks (Figure 1). We synthesize a series of copolymers comprising crosslinkable, high‐*T*_g_ poly(vinyl cyclohexene carbonate) (PVCHC, A block) and non‐crosslinkable, low‐*T*_g_ poly(propylene carbonate) (PPC, B block) arranged in ABA, BAB, and statistical sequences, and we photocure them in two selective solvents that preferentially swell either A or B. By combining photorheology of the printing resins with SAXS of both non‐crosslinked and crosslinked polymers, we show that block sequence and solvent‐selective swelling jointly regulate the accessibility and local environment of pendant vinyl groups during curing, thereby controlling gelation behavior and the extent of trapped microphase separation. These differences in network topology translate directly into mechanical response: when the crosslinkable segments are the outer blocks and cured in an A‐selective solvent, the printed networks exhibit markedly higher toughness at comparable modulus, whereas unfavorable combinations of sequence and solvent (e.g., centrally‐sequestered A blocks cured in a B‐selective solvent) yield stiff but brittle materials. These results establish clear structure–processing–property relationships and introduce polymer sequence and solvent‐selective swelling as practical design knobs for tuning the mechanical properties of crosslinked BCPs for additive manufacturing.

## 2 Results and Discussion

### 2.1 Synthesis, Characterization, and Resin Formulation

To directly probe the role of polymer sequence, ABA, BAB, and statistical (Stat) copolymers were prepared via controlled ring‐opening copolymerization (ROCOP) of propylene oxide (PO) and 4‐vinylcyclohexene oxide (VCHO) with CO_2_ [24]. This epoxide monomer pair affords a classical hard/soft motif: at room temperature, the PVCHC block is glassy (denoted as A block, *T*_g_ ≈ 110°C), whereas the PPC block is soft (denoted as B block, *T*_g_ ≈ 30°C) [24]. The PVCHC and PPC homopolymers were also synthesized for comparison [24, 26]. Importantly, only VCHC units possess pendant vinyl groups required for thiol–ene crosslinking with 1,6‐hexanedithiol under UV irradiation in the presence of a photoinitiator. Full procedures and characterization of all polymers are provided in the Experimental Section and Supporting Information (Figures S1–S13).

**FIGURE 2:**
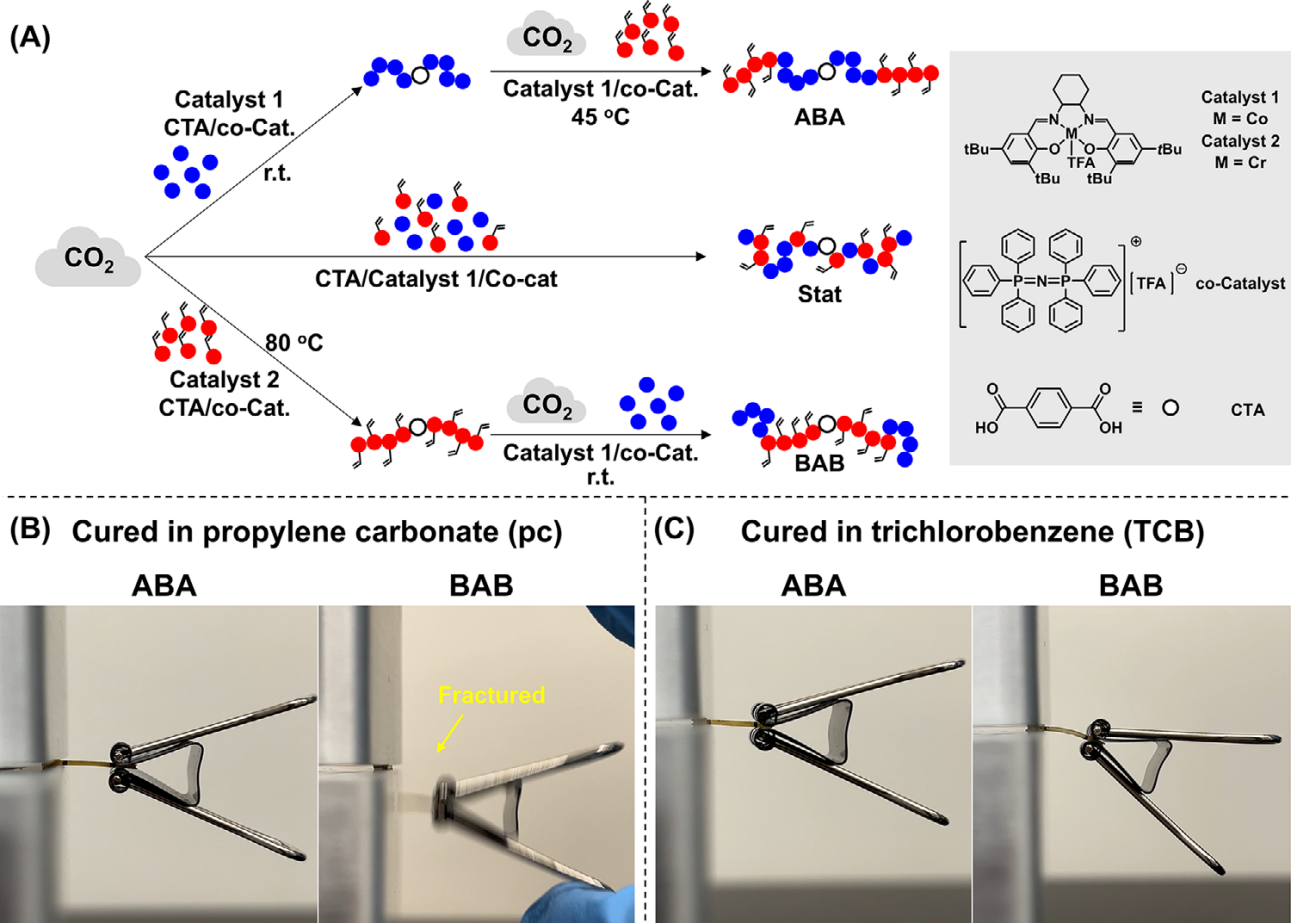
Sequence‐controlled synthesis and solvent‐dependent toughness in polycarbonate networks. (A) Schematic of controlled ROCOP of PO and VCHO with CO_2_ to access ABA, BAB, and statistical copolymers. (B) and (C) Qualitative toughness comparison of thiol–ene–crosslinked (1,6‐hexanedithiol) ABA and BAB networks cured in propylene carbonate or in 1,2,4‐trichlorobenzene, revealing pronounced solvent‐ and sequence‐dependent resistance to fracture. Cantilever specimens (≈10 mm × 2 mm × 0.66 mm) were loaded with a metal clip (≈7.7 g).

As shown in Figure 2A, all copolymers were prepared to match targeted weight‐average molar mass (*M*_w_), A:B ratio, and dispersity (*Đ*). Briefly, statistical copolymers were obtained by charging both epoxide monomers together with (salen)Co(III)TFA and PPNTFA into a stainless‐steel reactor under an inert atmosphere, then pressurizing with CO_2_; copolymerization was carried out at room temperature. ABA triblocks were synthesized in one pot via a two‐step sequence. First, PO was polymerized from a terephthalic acid chain‐transfer agent (CTA) using (salen)Co(III)TFA/PPNTFA to form the central, telechelic PPC. After complete PO consumption, VCHO and CO_2_ were introduced to grow PVCHC blocks from both ends of PPC, affording PVCHC–PPC–PVCHC (ABA). The same one‐pot/two‐step approach was unsuccessful for BAB: in the presence of terephthalic acid, VCHO/CO_2_ insertion to PVCHC is slow and requires elevated temperature, at which the Co catalyst is thermally unstable, leading to zero VCHO conversion. To access BAB, we therefore implemented a tandem protocol: a more thermally robust (salen)Cr(III)TFA/PPNTFA was used at 80°C to generate the telechelic PVCHC [27, 28]; subsequently, (salen)Co(III)TFA, PO, and CO_2_ were introduced at room temperature to grow PPC from both ends of PVCHC, yielding the inverted PPC–PVCHC–PPC (BAB). Here, the residual Cr catalyst remained dormant under the Co‐catalyzed conditions, enabling a one‐pot, two‐metal (Cr/Co) process mediated by the shared cocatalyst PPNTFA. As shown in Figures S1 and S2, size exclusion chromatography (SEC) and ^1^H nuclear magnetic resonance (NMR) spectroscopy monitoring during the preparation of ABA and BAB validated the block architectures. The A:B ratios calculated from ^1^H NMR were consistent across samples (1:0.96 to 1:0.99), corresponding to ∼3.6 mmol g^−1^ vinyl groups (see Supporting Information, Equation S1, for details). SEC (Figures S3–S5) confirmed *M*_w_ values of 19.0–21.4 kDa for all copolymers with narrow dispersities (*Đ* = 1.08–1.12). This consistency ensures that differences in performance arise from sequence effects rather than composition.

The copolymers were used to prepare crosslinked specimens for subsequent mechanical testing and characterization by x‐ray scattering. Based on the estimated vinyl content, an alkene:thiol molar ratio of 1:0.4 was chosen to minimize unreacted thiols and to ensure comparable crosslink density across samples using 1,6‐hexanedithiol as the thiol crosslinker. Polymers were dissolved at 300 mg mL^−1^ in either propylene carbonate (PC) or 1,2,4‐trichlorobenzene (TCB) with 0.5 wt% DMPA (photoinitiator) relative to total mass; this solution was drop‐cast onto a glass slide and UV‐irradiated for 90 s (30 s each on the top, bottom, and sides). The resulting films were washed with toluene, toluene/isopropanol mixture, ethyl acetate, and acetone and vacuum‐dried, as detailed in the Experimental Section; FTIR spectroscopy and thermogravimetric analysis (TGA) confirmed solvent removal; FTIR showed residual alkene signals at 1640 cm^−1^ as expected for the given alkene:thiol ratio (Figures S13 and S14).

Surprisingly, despite negligible differences in FTIR profiles after drying, the samples made from isomers ABA and BAB displayed contrasting solvent‐dependent mechanical responses. Crosslinked ABA networks (xABA) remained tough regardless of curing solvent (Figure 2B,C, left), whereas xBAB exhibited a pronounced solvent effect: samples cured in PC were brittle and fractured readily (Figure 2B, right), whereas those cured in TCB showed markedly higher toughness (Figure 2C, right). These results imply that both polymer sequence and solvent selectivity may affect the network formation during photocuring, which in turn dictates the bulk mechanical toughness and failure mode of the material.

### 2.2 Polymer–Solvent Interactions and Photocuring Kinetics

**FIGURE 3:**
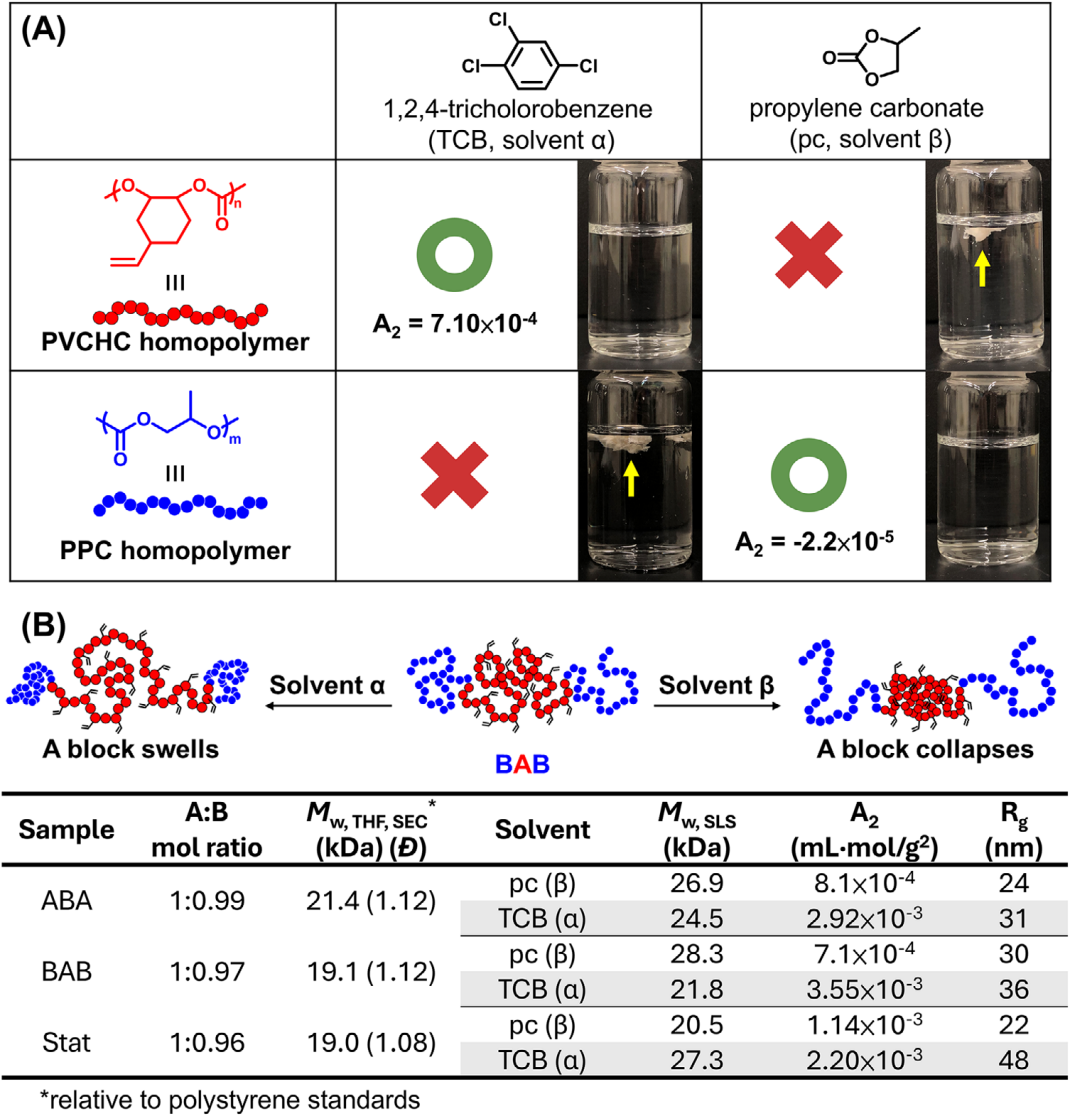
(A) Selective solubility of PVCHC and PPC homopolymers: photographs and second virial coefficients (A_2_) determined by static light scattering (SLS). (B) Schematic cartoons of the expected solvation behavior of BAB triblocks, and the copolymer information obtained from THF SEC and SLS.

To understand the polymer–solvent interactions, we first examined the solvation of PVCHC and PPC homopolymers in the dilute/semidilute regime by static light scattering (SLS) (Figure 3A, Figures S15 and S16). The second virial coefficient (*A*_2_) calculated using the Zimm formalism quantifies polymer‐solvent interactions, where *A*_2_ > 0 indicates a good solvent, *A*_2_< 0 a poor solvent, and *A*_2_ = 0 θ conditions [29]. Based on the A_2_ values and visual determination of solubility (Figure 3A), we found that TCB is a good solvent for PVCHC homopolymer, but it does not dissolve PPC homopolymer; in contrast, PC is a theta solvent of PPC but it does not dissolve PVCHC. Additional solvent screening (dimethylformamide, acetone, tetrahydrofuran, ethyl butanoate, and ethyl acetate) showed PPC aggregation above ∼5 mg mL^−1^, making PC the only solvent that maintained PPC in a homogeneous solution over the concentration range of interest. Therefore, we believe that this solvent pair enables selective solvation of one of the blocks while rendering the other block poorly solvated, as illustrated in Figure 3B (top). We denoted TCB as solvent *α* as it preferentially solvates PVCHC (A); similarly, PC is denoted as solvent *β* as it preferentially solvates PPC (B).

We then performed SLS on ABA, BAB, and Stat copolymers (Figure 3B, bottom; Figures S17–S22). Across concentrations of 0.6–10 mg mL^−1^, the molar masses (*M*_w_) from SLS closely matched the SEC values, supporting that the copolymers are dissolved as individual polymer chains under these dilute‐to‐semidilute conditions. Consistent with the homopolymer screening, *A*_2_ values of all polymers were higher in solvent α than in solvent *β*, indicating modestly improved solvation in TCB, as expected: solvent α is a good solvent for half the polymer, whereas solvent *β* is a theta solvent. *R*_g_ values are included in Figure 3B; however, these values for low molar mass polymers fall near the lower detection limit of Zimm analysis and are therefore not interpreted quantitatively. Taken together, these measurements support that all copolymers are solvated in both solvents and do not significantly aggregate at low conditions. This suggests that any curing differences discussed are not caused by simple solubility but are more likely governed by the sequence‐ and solvent‐dependent accessibility of vinyl units under concentrated, curing‐relevant conditions.

Indeed, dilute‐solution behavior does not necessarily predict curing at processing‐relevant concentrations. To understand how the accessibility of the reactive A block governs crosslinking, we investigated network formation as a function of concentration and identified the critical gelation concentration (CGC) for each resin. Resins were formulated as described above but with varied polymer concentrations (25–150 mg mL^−1^) and photorheology was performed to determine the concentration at which the storage and loss moduli first cross over during photocuring.

**FIGURE 4:**
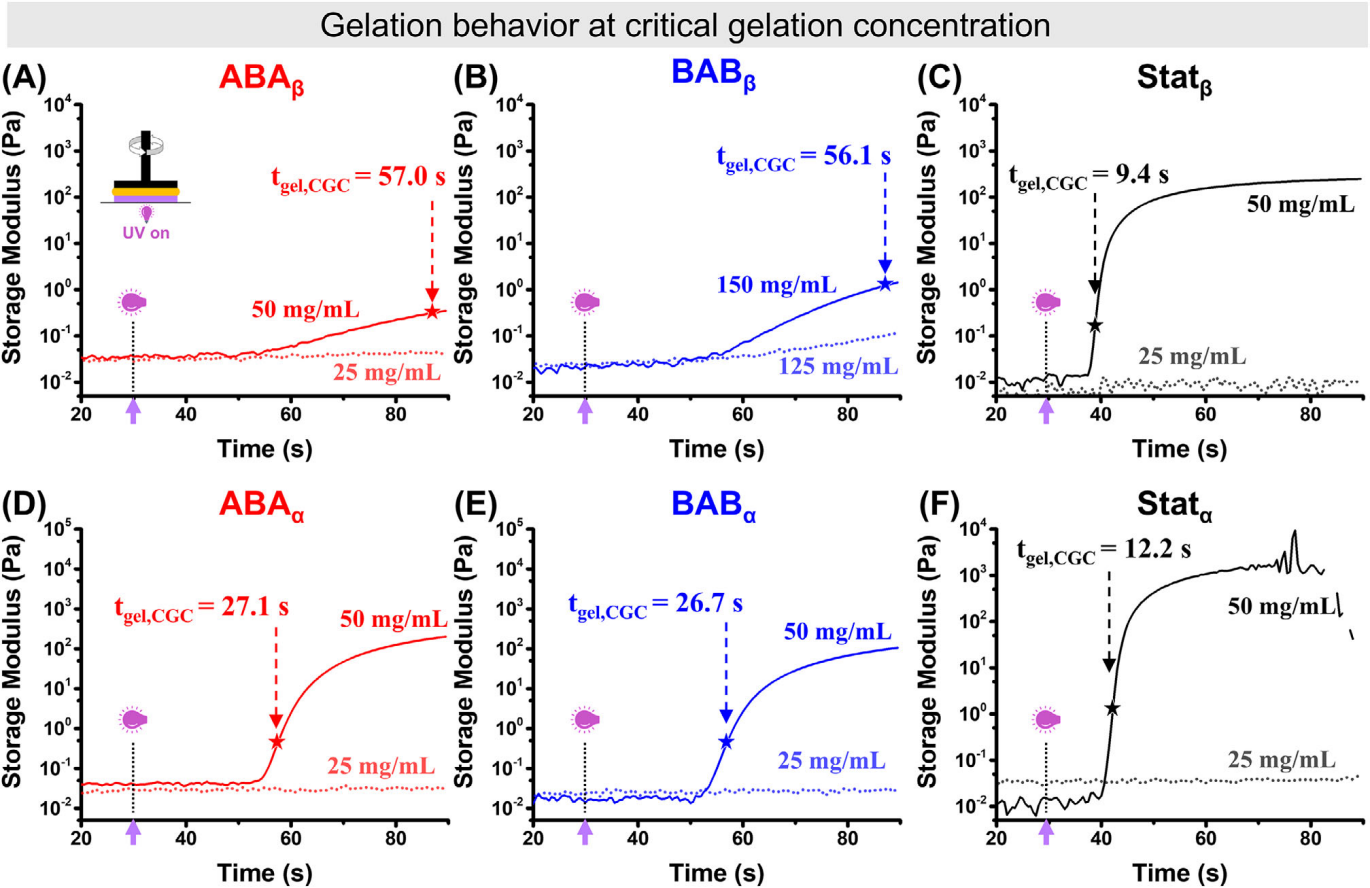
Critical gelation concentration characterization of ABA, BAB, and Stat resins at varying concentrations in solvent *β* (A–C) and solvent *α* (D–F). Critical gelation times (*t*_gel,CGC_), when the storage modulus crosses over the loss modulus, are indicated. Concentrations are noted in each panel.

**FIGURE 5:**
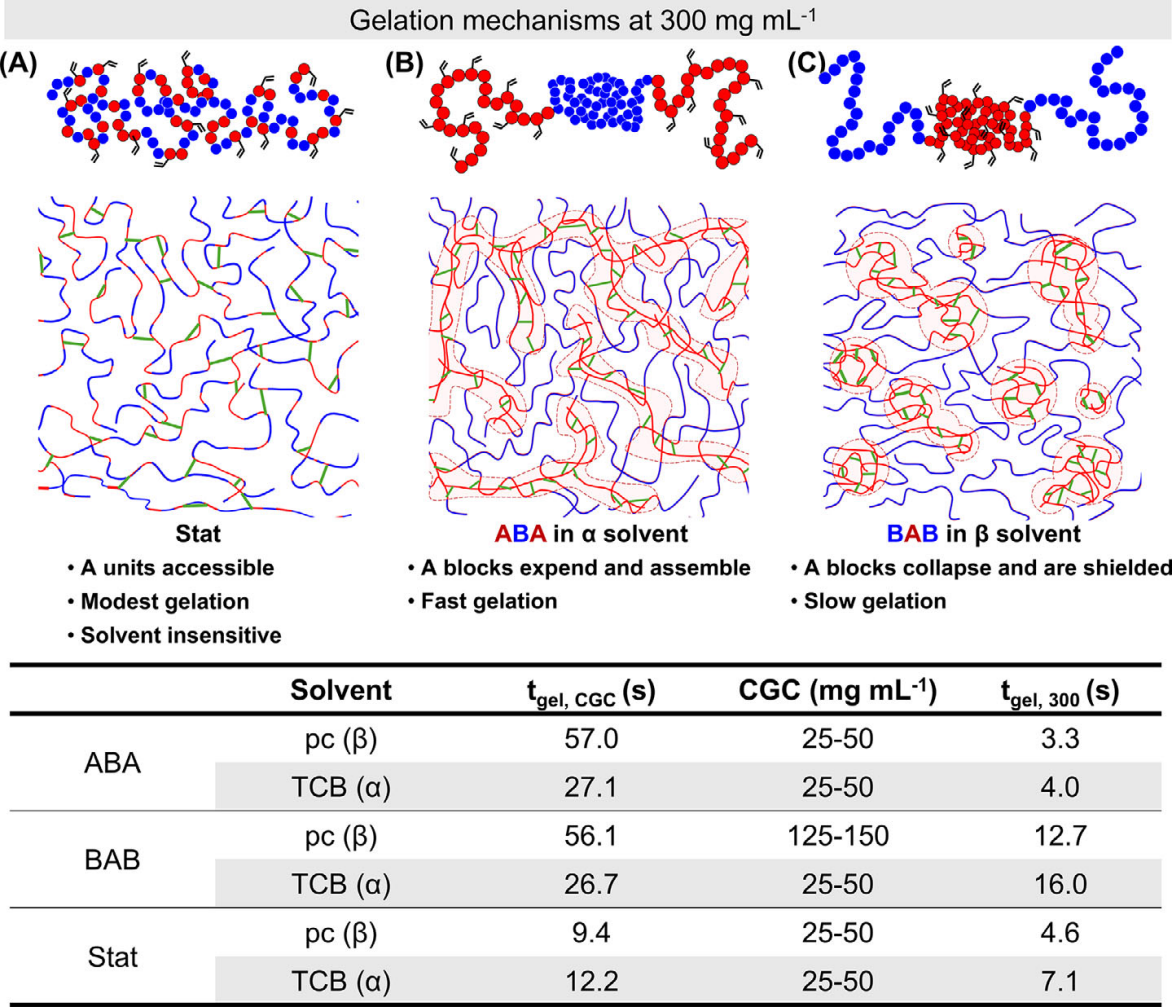
Schematic illustration of sequence‐dependent gelation in representative solvents and summary of photorheology results for ABA, BAB, and Stat resins in *β* and *α* solvents. (A) Stat crosslinks uniformly in both solvents, (B) ABA in solvent *α* promotes network formation, and (C) BAB in solvent β hinders network formation.

As shown in Figures 4 and 5, Stat gelled fastest in both solvents at concentrations slightly above the CGC. This can be attributed to the homogeneously distributed reactive units in the solution. In the Stat, the VCHC units are randomly distributed along the chain and thus lack microphase separation. Once the thiol‐ene crosslinking reaction is initiated, the chains are locked immediately due to the quick formation of the network. The UV attenuation effect of the solvents also slightly alters the gelation time; therefore, Stat cures faster in solvent *β* (PC, more UV‐transparent) than in solvent *α* (TCB, an aromatic solvent that absorbs UV). In contrast, the BCPs can undergo phase separation, causing effective gelation to require A‐rich domains to come into spatial proximity (inter‐domain contact). In both ABA and BAB, B blocks segregate into B‐rich regions that isolate A‐rich PVCHC domains; thus, reaching the gel point requires sufficient crosslinking within the A domains (longer gelation time) to immobilize the entire network. This constraint is even stronger in solvent *β*, where *A* blocks are more compact, thereby screening *A* domains from one another and preventing inter‐domain contact. Consequently, ABA_β_ and BAB_β_ (resins prepared in solvent *β*) gel more slowly than their α counterparts at the CGC, and BAB_β_, whose *A* domains in each chain are isolated by outer *B* blocks, requires a substantially higher polymer concentration (125–150 mg mL^−1^) than the other samples (25–50 mg mL^−1^). This behavior emphasizes that even polymers with comparable dilute‐solution dimensions can display markedly different gelation behavior due to sequence‐ and solvation‐tuned accessibility of their crosslinkable domains. Only when reactive segments form a continuous network can the BCP solution be readily photocured.

We next examined curing kinetics at a concentration relevant to vat polymerization (300 mg mL^−1^) using photorheological time sweeps (Figure 5, Figure S23). At this concentration, all resins are well above their CGC and form effective networks, so gelation is significantly faster and strongly affected by solvent UV attenuation. Notably, ABA reaches the gel point fastest in both solvents; this indicates that self‐assembly becomes decisive at high concentration, as previously reported in polyurethane networks [30]. As illustrated graphically in Figure 5B, A‐rich aggregates in ABA bridge chains and locally concentrate intra‐ and interchain vinyl groups, thereby accelerating crosslinking. In BAB (Figure 5C), the B blocks interrupt the formation of extended A domains, yielding centrally trapped A‐rich domains, which suppresses terminal bridging and limits inter‐domain connectivity. As a result, the crosslinking of BAB remains localized and takes longer to immobilize the free chains even at high concentration (Figure S23). Consistent with this picture, we also evaluated an additional, higher‐molar‐mass BAB sample (*M*_w,SEC_ ≈ 40 kDa; A:B = 1:1) in solvent β, which failed to form continuous films and instead yielded fragmented, uneven objects (Figure S24), reflecting a mechanically fragile, poorly percolated network in which thicker B domains more effectively shield the A domains.

### 2.3 Microphase Morphology Analysis

Moving from the solution state to dried solid samples, solution‐cast films were washed and vacuum‐dried as described in the Experimental Section, and small‐/wide‐angle x‐ray scattering (SAXS/WAXS) was used to probe the morphology of the copolymers before and after crosslinking. These measurements were designed to answer three mechanistic questions: whether the copolymers form nanoscale microphase‐separated structures, whether photocrosslinking preserves or disrupts these structures, and whether the trapped morphologies remain stable under tensile deformation. In SAXS, the position of the primary scattering peak, Q*, provides the characteristic real‐space spacing of nanoscale domains, while the peak width reflects the degree of spatial correlation or distribution of domain spacings. The peak intensity is related to the electron‐density contrast and the extent of segregation between A‐ and B‐rich regions. WAXS, by contrast, probes shorter‐range atomic or segmental packing and was used here to rule out crystallinity as a contributor to the observed mechanical differences. Thus, this scattering analysis provides a direct structural basis for linking polymer sequence and curing solvent to the network morphology responsible for the observed toughness trends.

**FIGURE 6:**
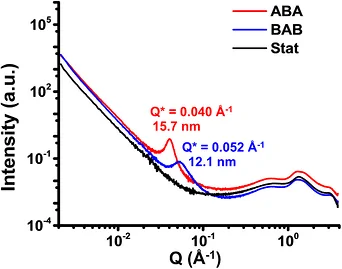
SAXS of uncrosslinked ABA, BAB, and Stat.

As shown in Figure 6, WAXS profiles (*Q* > 0.25 Å^−1^) of all uncrosslinked samples show only a broad amorphous hump centered at *Q* ≈ 1.4 Å^−1^, corresponding to an average backbone spacing of approximately 4.5 Å. The absence of sharp Bragg reflections confirms that these materials are amorphous at the atomic scale,[31] indicating that the structural differences discussed below arise from nanoscale composition fluctuations rather than crystallinity. In the SAXS region (*Q* < 0.25 Å^−1^), both uncrosslinked ABA and BAB (pellets dried from DCM) exhibit a single, relatively broad primary peak (Δ*Q*/Q* ≈ 0.21–0.29, where Δ*Q* is the full width at half maximum), consistent with a poorly ordered microphase‐separated state characterized by enhanced A/B composition fluctuations and only short‐range correlations. The absence of higher‐order peaks rules out long‐range ordered morphologies. In contrast, uncrosslinked Stat displays no SAXS peak, indicating an essentially homogeneous structure on the 1–100 nm length scale. Notably, ABA exhibits a lower Q* and a sharper primary peak than BAB (Δ*Q*/Q* = 0.20 vs. 0.30, respectively), indicating a larger characteristic spacing and stronger short‐range order (i.e., nanoscale A‐ and B‐rich regions are more regularly spaced over short distances). The differences in peak position and width between ABA and BAB are attributed to segmental asymmetry and architectural constraints. The A block possesses greater segmental rigidity (estimated from *T*_g_) and nearly twice the molar volume of the B block (see Experimental Section), which biases A‐rich composition fluctuations toward lower interfacial curvature relative to B‐rich regions.[32, 33] In ABA, a compliant B midblock connects two rigid A outer blocks, increasing the likelihood of interdomain A–A connectivity through tie‐chain–like configurations. This enhanced connectivity promotes more correlated spacing between neighboring A‐rich regions, which appears in SAXS as a lower Q* value, corresponding to a larger characteristic spacing, and a sharper primary peak. In contrast, in BAB the rigid A block is centrally confined between two soft B blocks, suppressing A–A contacts and limiting the development of correlated A‐rich fluctuations. This architectural confinement leads to weaker segregation and a broader, lower‐intensity SAXS peak, consistent with a reduced correlation length and a wider distribution of composition fluctuations. This trend is also consistent with the photorheology results discussed above.

**FIGURE 7:**
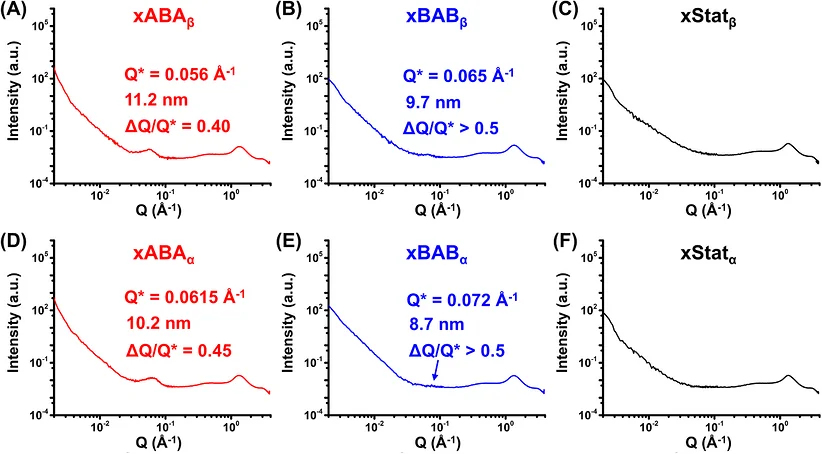
SAXS profiles of crosslinked block copolymer networks. (A, D) xABA, (B, E) xBAB, and (C, F) xStat prepared in solvent β (top) and solvent α (bottom).

After establishing the baseline morphology of the uncrosslinked copolymers, we next examined how photocrosslinking and solvent removal altered these nanoscale structures. In the SAXS profiles of photocrosslinked samples (Figure 7), the main SAXS peaks of xABA and xBAB shift to higher *Q* (from ∼0.04–0.05 to ∼0.06–0.07 Å^−1^) and decrease significantly in intensity, indicating a reduced characteristic spacing (∼12–16 nm to ∼9–11 nm) and poorer contrast between domains. We attribute this reduction to solvent screening during photocrosslinking in the solution state: in concentrated, highly swollen solutions, strong chain interpenetration partially screens the effective *χ*_AB_, reducing the driving force for phase separation and domain coarsening. Rapid UV curing locks this early‐stage structure and limits further coarsening during drying due to network constraints, thereby confining microphase separation to shorter length scales. Among the crosslinked BCPs, xABA retains a distinct, higher‐intensity, and narrower peak (Δ*Q*/Q* ≈ 0.40–0.45), indicating more pronounced short‐range order, whereas xBAB shows only an extremely weak, broad feature near the background (Δ*Q*/Q* > 0.5, estimated), consistent with weaker segregation and a broader distribution of domain spacing.

To distinguish whether the photocrosslinked morphology was permanently fixed or could further reorganize upon thermal activation, we performed in situ heating SAXS above the *T*_g_ of both blocks (Figure S25). Upon annealing, the SAXS peak intensity increased, and the normalized peak width decreased (Δ*Q*/Q* ≈ 0.30 for xABA and 0.27 for xBAB), while Q* remained essentially unchanged. This result indicates that thermal activation enhances short‐range order without changing the characteristic domain spacing. We hypothesize that, because these materials form covalently crosslinked networks, the topological constraints imposed by crosslinking prevent domain coarsening even when thermal mobility increases (Q* remains unchanged). At the same time, because the thiol:vinyl ratio (0.4:1) limits the crosslinking conversion to ∼40% at most, the network remains flexible when heated above the *T*_g_ of both blocks; thermal cycling can therefore enhance short‐range order (decreasing Δ*Q*/Q*) by allowing non‐crosslinked segments (*B* blocks and uncrosslinked *A* blocks) and interfacial chains to reorganize and repartition within the semi‐fixed morphology, and thus effectively increase the apparent segregation and sharpen the SAXS peak. In contrast, xStat remains essentially featureless before and after annealing, confirming a single‐phase morphology and that heating/cooling cycle does not induce microphase separation in a fully disordered system.

**FIGURE 8:**
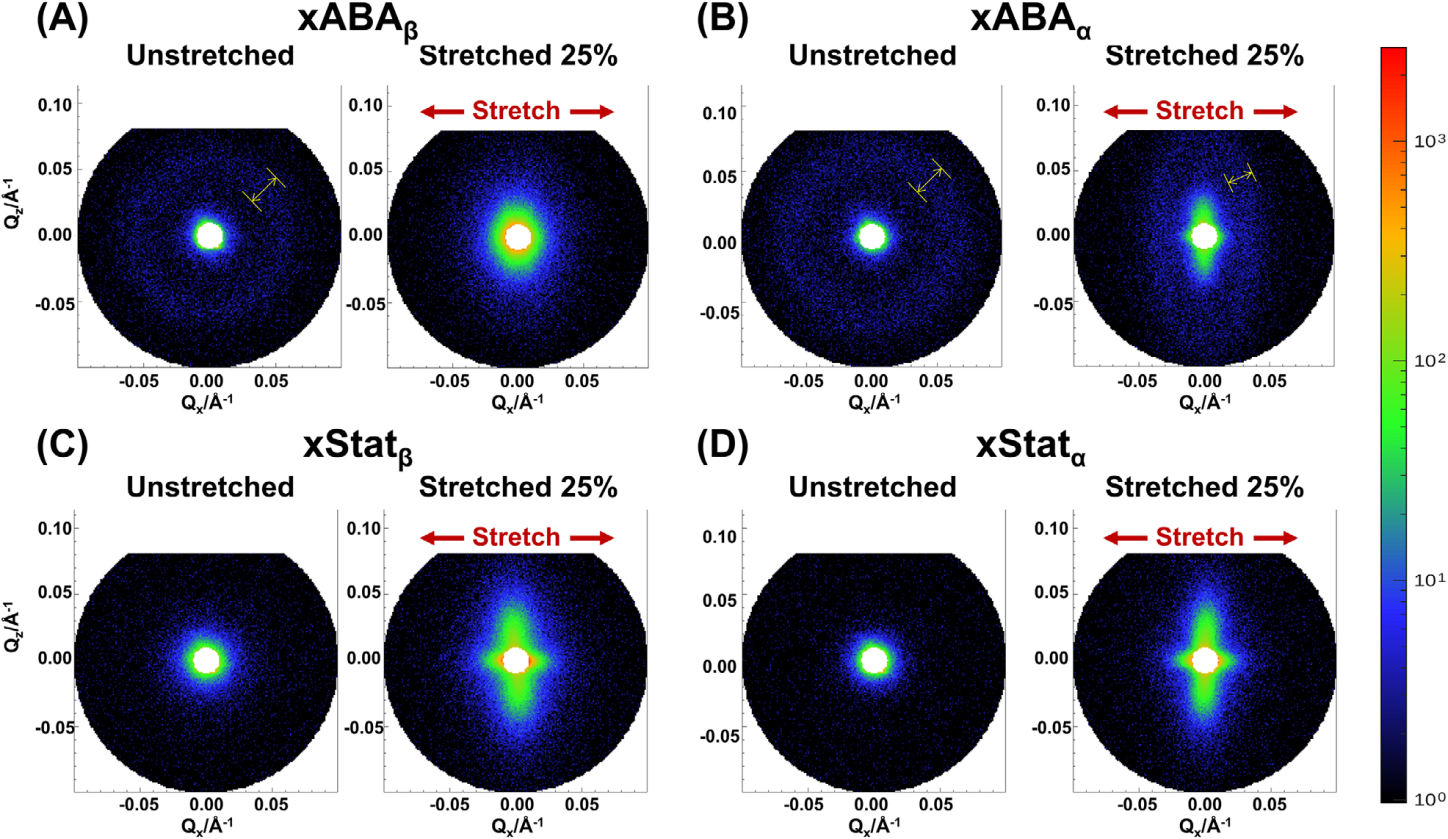
2D SAXS patterns of (A) xABA_β_, (B) xABA_α_, (C) xStat_β_, and (D) xStat_α_ before and after uniaxial stretching for 5 mm (∼25% strain).

To directly connect the trapped morphology to mechanical deformation, we performed in situ tensile SAXS at 40°C. Changes in the 2D scattering pattern under strain reveal whether nanoscale A/B correlations are preserved, oriented, or disrupted, thereby providing structural insight into how each network accommodates and dissipates mechanical energy. As shown by the 2D SAXS patterns in Figure 8, in the unstretched state, both xABA_α_ and xABA_β_ exhibit an isotropic ring at Q* ≈ 0.06 Å^−1^, consistent with the ∼10 nm microphase‐separated morphology described above. Upon uniaxial stretching, xABA_β_ rapidly loses this ring feature and approaches a diffuse halo, suggesting a strong reduction in A/B correlation and substantial disruption of the microphase‐separated morphology under strain. In contrast, xABA_α_ retains a pronounced scattering peak that evolves from an isotropic ring to an anisotropic, indicating that the underlying microphase‐separated morphology remains intact while undergoing orientation and deformation. This reflects how different crosslinked networks dissipate energy differently. When cured in solvent α, A‐rich regions are more swollen and interpenetrated, favoring a more continuous and uniformly crosslinked network (Figure 5B) that accommodates strain cooperatively. When cured in solvent β, A blocks collapse with limited interpenetration between neighboring A‐rich domains, leading to localized crosslinking, poor interdomain connectivity, and a mechanically fragile network (Figure 5C). Consequently, nanoscale A/B correlations are more readily destroyed under load. For xBAB_β_, post‐stretch 2D patterns could not be collected due to its brittleness (Figure S26A), but we believe that the same framework described above also applies. At high strains, xABA_α_, xStat_α_, and xStat_β_ develop enhanced low‐Q scattering with a cross‐ or butterfly‐like anisotropy (Figure 8B–D). These features occur at Q well below the microphase peak, corresponding to length scales of tens to hundreds of nanometers. We attribute these features to large‐scale strain heterogeneity and possible microvoid formation, consistent with prior reports on stretched polymer networks [34, 35, 36, 37]. The cross‐shaped anisotropy suggests that these heterogeneities/microvoids are oriented by a resilient network under deformation. In less resilient networks (e.g., xABA_β_ and xBAB_α_), stretching disrupts the network and produces heterogeneity/microvoids in a more random, less oriented manner.

Taken together, the SAXS results demonstrate that repeat‐unit sequence is the primary determinant of the extent and length scale of microphase separation, with ABA exhibiting the strongest and most persistent short‐range correlations, BAB displaying weaker and more weakly correlated fluctuations, and the statistical copolymer remaining effectively homogeneous (ABA > BAB >> Stat). Photocrosslinking kinetically traps these sequence‐dependent morphologies, suppressing post‐cure coarsening and fixing the characteristic length scale, while subsequent thermal annealing sharpens the SAXS features without shifting Q*, consistent with enhanced segregation within a topologically constrained network. Importantly, although polymer architecture largely sets the intrinsic spacing of the microphase‐separated structure, the curing solvent plays a critical role in defining network topology by regulating A‐block accessibility and A‐A connectivity during crosslinking. This solvent‐controlled network formation directly governs the stability of nanoscale correlations under mechanical deformation, as evidenced by the distinct tensile‐SAXS responses, thereby linking solvent conditions, trapped morphology, and mechanical performance.

### 2.4 Mechanical Performance and 3D Printing Demonstrations

**FIGURE 9:**
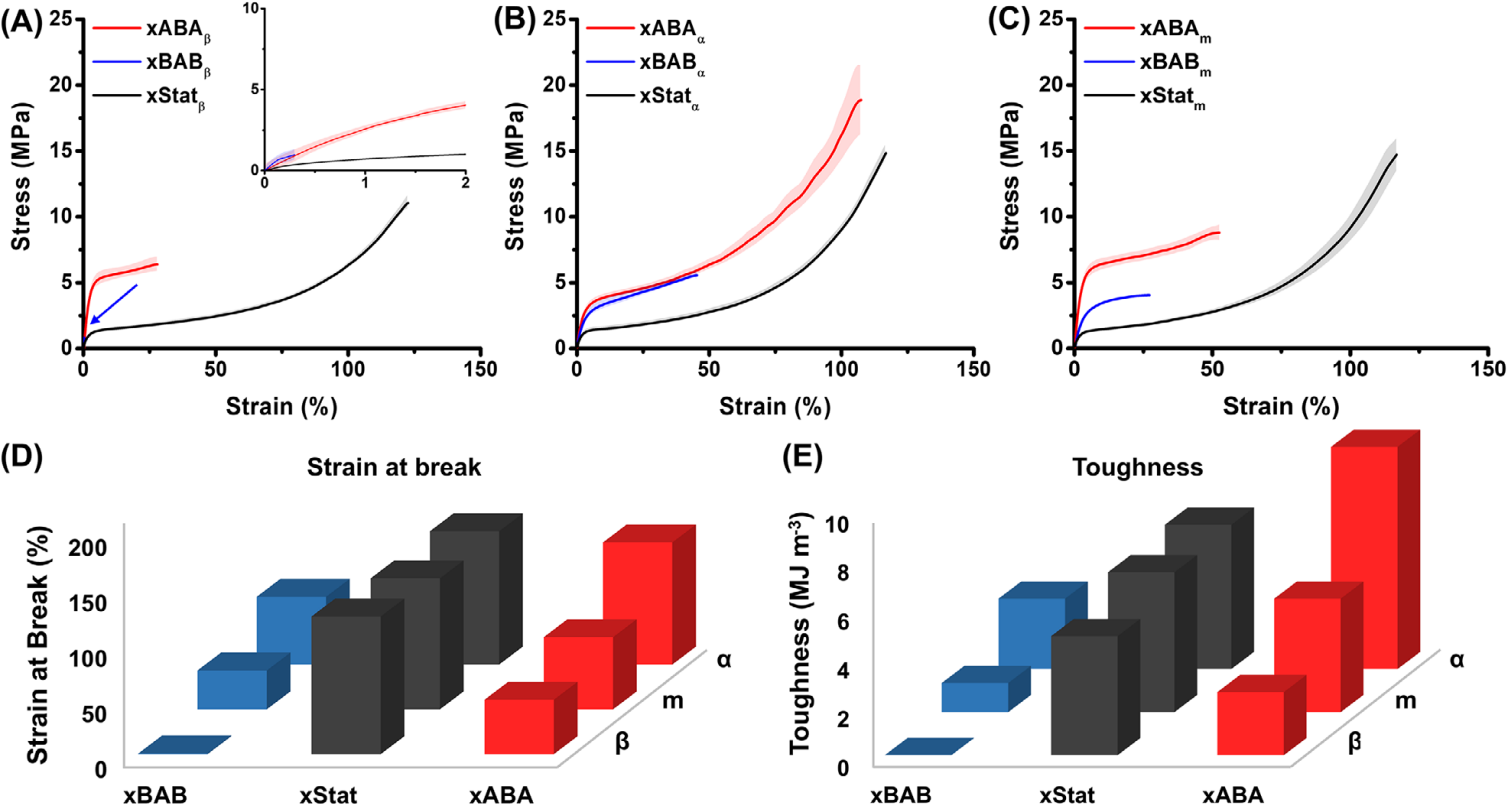
Representative tensile stress–strain curves of ABA, BAB, and Stat copolymers: (A) solvent *β*, (B) solvent *α*, and (C) mixed solvent of *α*:*β* (3:1 by volume). Summary maps of (D) strain at break and (E) toughness for ABA, BAB, and Stat cured in solvent *β*, *α*:*β* (3:1 by volume), and solvent *α*.

Having elucidated the key differences in network formation and microphase separation morphology, we next examined how these differences translate to bulk mechanical performance. To this end, we prepared 20 mm × 3 mm × 1 mm tensile bars from networks cured in solvent *β*, solvent *α*, and a mixed solvent (*α*:*β* = 3:1 by volume), and measured their tensile properties to demonstrate property modulation based on solvent ratio. Tensile properties are summarized in Table S1, and representative stress–strain curves are shown in Figure 9A–C.

Across all solvent formulations, xStat exhibits the lowest tensile modulus, consistent with its essentially featureless SAXS profiles and the absence of microphase‐separated domains that would reinforce the network. Meanwhile, xStat displays the highest strain at break, consistent with uniformly distributed crosslinks throughout the network that are resilient to deformation, as supported by tensile‐SAXS measurements. For the BCP networks, sequence primarily dictates whether crosslinkable A segment can form interdomain bridges: in xABA, the outer A blocks readily bridge between A‐rich domains, promoting interdomain connectivity and more cooperative load sharing; in xBAB, the centrally confined A block is more effectively shielded by surrounding B domains, biasing crosslinking toward localized (intradomain) A‐rich regions and limiting interdomain connectivity. Consistent with these connectivity arguments, xABA outperforms xBAB in ultimate strength, strain at break, and toughness in every solvent where rigorous tensile testing is feasible. Notably, xBAB develops macroscopic drying cracks in solvent α and the mixed solvent (Figure S27), likely due to greater internal stress within localized A‐rich domains; this precludes quantitative comparison across sequences under those conditions.

Superimposed on this sequence effect, solvent tunes A‐domain accessibility during curing: in solvent *β*, *A* domains collapse and suppress interdomain contacts, producing more locally crosslinked, internally stressed domains that limit extensibility and promote premature fracture (Figure 5C); this is reflected in increased modulus and decreased strain at break. As the fraction of solvent α increases, A‐rich domains become swollen and interpenetrated, favoring interdomain crosslinking and a resilient morphology that can orient and deform under load (Figure 5B), resulting in increased toughness, strain at break, and ultimate strength. The mixed solvent yields intermediate behavior that offers a practical stiffness–ductility compromise. Finally, xStat remains comparatively solvent‐insensitive as its vinyl groups remain uniformly accessible regardless of solvent, and the network lacks microphase‐separated reinforcement. In contrast, xBAB shows the strongest solvent response, with toughness increasing from 0.003 MJ m^−3^ in solvent *β* to 2.9 MJ m^−3^ in solvent *α* (≈10^3^‐fold).

Taken together, these results further support the orthogonal tunability: sequence sets the baseline accessibility and connectivity of crosslinkable domains, while solvent‐selective swelling modulates whether crosslinking proceeds in a swollen, interpenetrated state (α) or a collapsed, localized state (*β*). More importantly, these coupled effects translate directly to bulk mechanical performance, enabling programmable mechanical performance spanning nearly three orders of magnitude in toughness. To summarize how sequence and solvent govern mechanical performance, we map toughness, strain at break, and tensile modulus across formulations in Figure 9D–E and Figure S28. From these comparisons, three key trends can be identified: (1) xBCPs consistently exhibit higher modulus than xStat owing to microphase separation. (2) The processing solvent dictates the balance between swollen and collapsed crosslinking; for xBAB, changing from solvent *β* to solvent *α* increases network connectivity and yields an ∼10^3^‐fold increase in toughness. (3) Sequence and solvent act together: when *A* segments are end‐exposed (ABA), solvent α promotes crosslinking across terminal boundaries and yields tough materials; when *A* segments are centrally confined (BAB), solvent *β* favors localized crosslinking, producing stiff, brittle networks. By contrast, ABA in solvent β and BAB in solvent α are antagonistic pairings that suppress toughness gains.

**FIGURE 10:**
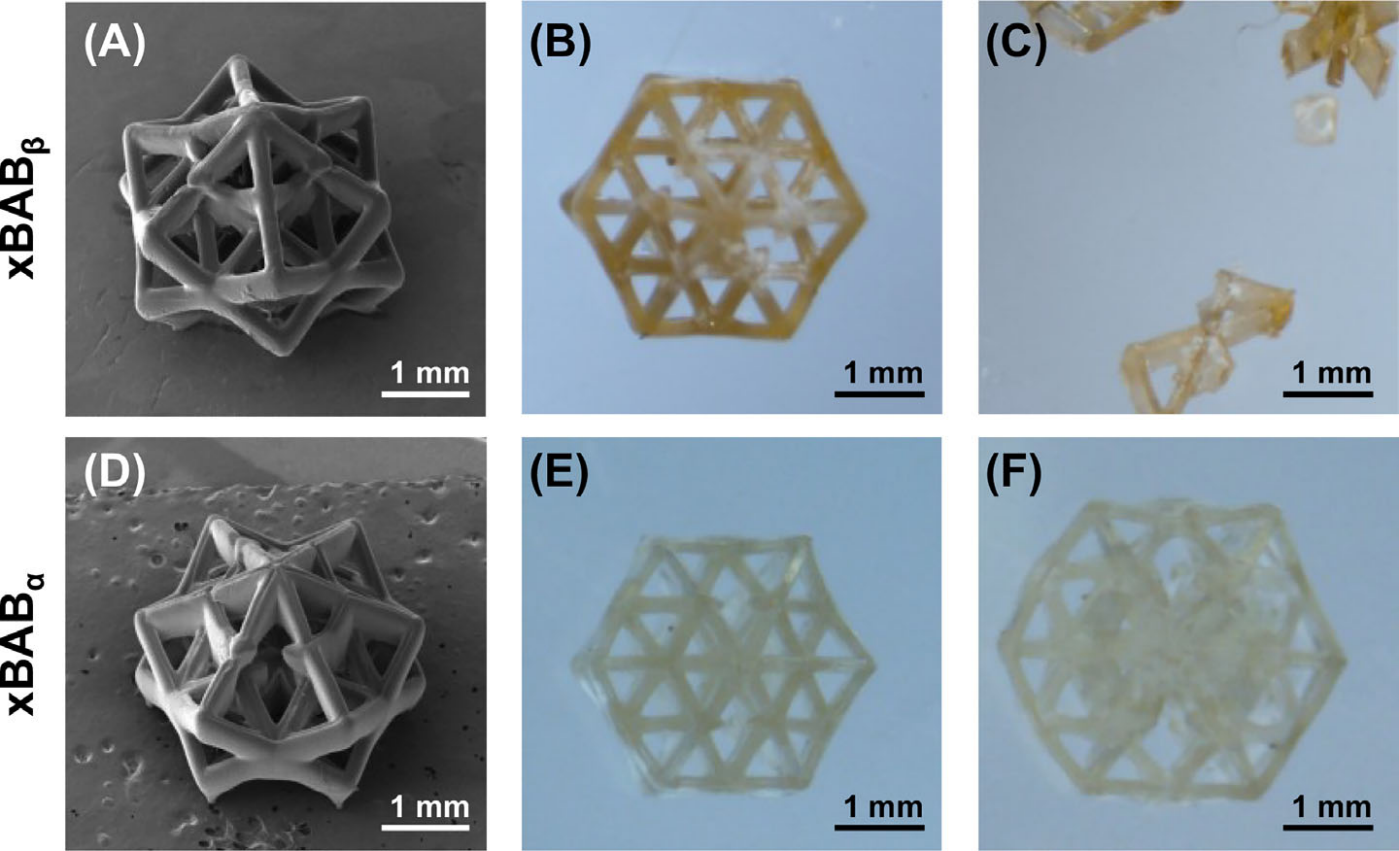
Mechanical behavior of dried, crosslinked BAB (xBAB) octahedrons 3D‐printed in solvents *β* and *α*. (A, D) SEM images of xBAB_β_ and xBAB_α_. (B, C) Optical microscope images of xBAB_β_ before and after compression, showing brittle fracture. (E, F) Optical microscope images of xBAB_α_ before and after compression, showing flexible deformation.

To demonstrate the practical implications of this multi‐knob tunability of mechanical properties in a printing context, we employed a lab‐built high‐resolution continuous liquid interface production (CLIP) setup to 3D print our resins [38]. As shown in Figure 10 and Figure S29, all three polymers formulated in solvents α and β were successfully printed. Consistent with the high resolution of CLIP, all printed objects exhibit smooth sidewalls. Notably, despite their different solvent formulations, both BAB resins (BAB_α_ and BAB_β_) were printed into identical octahedron lattices using the same parameters (Figure 10A,D and Table S2). Perhaps surprising, unlike the bulk‐cast samples that consistently cracked, the printed xBAB_α_ remained intact (Figure 10D). We propose that this success stems from the continuous growth at the oxygen‐inhibited “dead zone,” which minimizes internal stress buildup compared to traditional casting [39]. Consistent with the trends observed in the tensile tests, these lattices exhibited strikingly different mechanical responses under compression (Figure 10B,C,E,F, and Videos S1 and S2): the lattice printed in solvent *β* fractured (i.e., was brittle), whereas the lattice printed in solvent α deformed more flexibly with partial recovery upon unloading. Because the printing parameters were held constant, this confirms that the observed differences arise from solvent‐controlled network formation. These results underscore that controlling solvation to tune network topology provides a practical handle to program mechanical properties in additive manufacturing, enabling a single BCP resin platform to yield printed objects with distinct stiffness–toughness profiles.

## 3 Conclusions

In this work, we delineate how two independent design knobs, polymer sequence and solvent‐selective swelling, together enable orthogonal tuning of network formation and mechanical performance in photocrosslinked CO_2_‐based copolycarbonates for vat photopolymerization. Across ABA/BAB/statistical sequences at matched molar mass, composition, and thiol:vinyl ratio, the sequence of the polymer sets the baseline propensity for microphase separation and domain connectivity, while the curing solvent serves as a powerful, physical “switch” that controls the local environment and accessibility of the crosslinkable segments during network formation. Importantly, the independence of these two knobs enables practical control over both printability and mechanical performance. Through photorheology and SAXS experiments, we demonstrate that ABA architectures enable interdomain bridging via outer A blocks, whereas BAB sequences sequester reactive blocks centrally with uncrosslinkable B blocks, leading to more localized crosslinking and ineffective percolation. In addition, the use of an A‐favored solvent promotes crosslinking in a swollen, interpenetrated state, whereas the use of a B‐favored solvent collapses the A‐rich domains and suppresses inter‐domain A–A contacts and percolation, leading to a mechanical envelope of nominally identical formulations spanning over three orders of magnitude in toughness (0.003–9.1 MJ m^−3^). Finally, we demonstrate that this orthogonal tunability is actionable in additive manufacturing. Using a high‐resolution CLIP platform, we successfully printed all formulations and obtained mechanically distinct objects under identical print parameters, highlighting that microscale‐level control can translate to macroscale‐level performance. Collectively, these results establish clear structure–processing–property relationships and identify polymer sequence and selective solvation as powerful strategies for programming both printability and performance of BCP resins for additive manufacturing, guiding the development of next‐generation vat‐photopolymerizable materials.

## 4 Experimental Section

### 4.1 Materials

All reactions were carried out under argon in a glovebox. Glassware and stainless‐steel reactors were dried at 150°C for 24 h prior to use. Toluene and dichloromethane (DCM) were dried on an MBraun manual solvent‐purification system equipped with Alcoa F200 activated alumina desiccant. Propylene carbonate, methanol, 2,2‐dimethoxy‐2‐phenylacetophenone (DMPA), silver trifluoroacetate, 2,4,6‐trimethylbenzoyldiphenylphosphine oxide (TPO), terephthalic acid, and hydrochloric acid (37%) were obtained from Sigma‐Aldrich. High‐pressure carbon dioxide (CO_2_) (Praxair) was used as received. Bis(triphenylphosphine)‐iminium trifluoroacetate (PPNTFA) was purchased from BeanTown Chemicals. Propylene oxide (99%, isomeric mixture; Thermo Scientific) and 4‐vinyl‐1‐cyclohexene 1,2‐epoxide (98%, isomeric mixture; BeanTown Chemicals) were dried over CaH_2_ under reduced pressure prior to use. 1,6‐Hexanedithiol (97%) came from Alfa Aesar, and 1,2,4‐trichlorobenzene was obtained from Oakwood Chemical. (R,R)‐(−)‐N,N’‐Bis(3,5‐di‐tert‐butylsalicylidene)‐1,2‐cyclohexanediaminocobalt(II) [(salen)Co(II)] was supplied by AstaTech, and (salen)Cr(III)Cl was purchased from Strem.

### 4.2 Instrumentation

^1^H NMR spectra were recorded on a Bruker 400 MHz instrument using CDCl_3_ (δ = 7.26 ppm). Size‐exclusion chromatography (SEC) employed a Tosoh HLC‐8320 system, fitted with a refractive index (RI) detector and TSKgel Super HZ‐M columns, using tetrahydrofuran (THF) as the eluent and polystyrene standards for calibration. ATR‐FTIR spectra were collected on a Jasco FT/IR‐4600 with a diamond‐coated ZnSe crystal. Thermogravimetric analysis (TGA) was performed on a TA Instruments TGA 5500 under N_2_: samples were heated from ambient to 100°C at a rate of 20°C/min, held isothermally for 10 min, and then further heated to 600°C at the same rate. Mechanical testing was conducted as follows: Tensile tests were performed using a TA Instruments DMA 850 equipped with a film clamp, at a loading rate of 3 mm/min. Photorheology measurements were performed on a TA Instruments DHR‐2 rheometer using both a 25 mm quartz parallel plate and a 25 mm stainless steel upper parallel plate, with a fixed gap of 500 µm. UV irradiation was applied using a UV LED (0.5 mW cm^−2^), which was turned on at 30 s, and the test continued for an additional 60 s. Samples were characterized in oscillatory mode with a strain amplitude of 0.2% and a frequency of 1 Hz to monitor the storage and loss moduli before and after UV exposure. For critical gel concentration (CGC) tests, the strain amplitude was increased to 1%. Static light scattering (SLS) measurements were conducted using an ALV/CGS‐3 Compact Goniometer. The (dn/dc) values were measured at 25°C using a WYA‐3S ABBE digital refractometer. Polymer solutions (0.6–10 mg mL^−1^) were prepared 24 h in advance and filtered twice through 0.2 µm PTFE filters prior to the measurements. Scattering data were collected over an angular range of 30° to 150°. The second virial coefficient (A_2_), radius of gyration (*R*_g_), and absolute weight‐average molecular weight (*M*_w_) were determined from Zimm plot extrapolation. Scanning electron microscopy (SEM) was conducted using a JEOL JSM‐7500F SEM. Prior to imaging, samples were coated with ∼10 nm Au.

### 4.3 Synthesis of (Salen)Co(III)TFA and (Salen)Cr(III)TFA

(salen)Co(II) or (salen)Cr(III)Cl was dissolved in dry DCM under N_2_. One equivalent of silver trifluoroacetate was added, and the mixture was stirred for 6 h. The resulting suspension was filtered, and the filtrate was evaporated under N_2_ to give dark‐green [(salen)Co(III)]TFA and brown [(salen)Cr(III)]TFA, respectively, which were used without further purification.

### 4.4 Synthesis of PPC‐b‐PVCHC‐b‐PPC (BAB)

In a stainless‐steel reactor, (salen)Cr(III)TFA (3.9 mg, 0.0056 mmol), PPNTFA (3.6 mg, 0.0056 mmol), 4‐vinyl‐1‐cyclohexene 1,2‐epoxide (VCHO, 0.75 mL, 5.6 mmol), and terephthalic acid (10.3 mg, 0.062 mmol) as a chain transfer agent (CTA) were mixed in toluene (0.4 mL) and DCM (0.4 mL) under argon. After pressurizing to 3 MPa with CO_2_, the mixture was stirred for 8–10 h to reach ≈100% conversion to poly(4‐vinyl cyclohexene carbonate) (PVCHC homopolymer). (Salen)Co(III)TFA (3.9 mg, 0.0056 mmol) and propylene oxide (PO, 0.39 mL, 5.6 mmol) were then introduced, the reactor was repressurized to 3 MPa with CO_2_, and the mixture was stirred at 24°C for 5–6 days. The progress of the reaction was monitored by NMR. The crude product was dissolved in DCM and precipitated in acidic methanol (repeated 4–5 times) to afford the pure BAB triblock copolymer.

### 4.5 Preparation of Crosslinked Cast Films

Copolymers were dissolved in propylene carbonate or 1,2,4‐trichlorobenzene (300  mg mL^−1^). 1,6‐Hexanedithiol (thiol:vinyl  =  0.4:1, calculated from *M*_n_ and mol % of VCHC in the polymer chain, see Equation S1) and DMPA (0.5 wt% relative to the total mass of the solvent, polymer, and thiol) were added. After sonication, the homogeneous mixture was cast on glass slides and irradiated with 365 nm UV light (∼1300 mW cm^−2^) for 30 s on each face (total 90 s). Films were sequentially washed: toluene (20 min), 1:1 toluene/isopropanol (20 min), ethyl acetate (2 × 5 min), and acetone (2 h). Samples were air‐dried for 10 min and dried under vacuum at room temperature for 48 h to ensure complete removal of residual solvents.

### 4.6 X‐Ray Scattering Experiments

All tests were performed under vacuum on a Xeuss 3.0 system (Xenocs, Grenoble, France). The instrument is equipped with a GeniX 3D x‐ray source providing monochromatic Cu Kα radiation (*λ* = 1.542 Å), and scattering patterns were collected with a Dectris Eiger 2R 1M‐pixel 2D detector. For room‐temperature (21°C) measurements of uncrosslinked and crosslinked copolymers, the sample‐to‐detector distance was set to 45, 370, 900 mm, and 1800 mm, which together provided an accessible q‐range of 0.002–3.5 Å^−1^. The scattering vector is defined as $Q=\left(\frac{4\pi }{\lambda }\right)\sin\frac{\theta }{2}$, where *θ* is the scattering angle between the incident and scattered beams. Two‐dimensional patterns were acquired in line‐eraser mode and reduced to one‐dimensional *I*(*q*) profiles after calibration using LaB_6_ and silver behenate standards. For variable‐temperature SAXS experiments, samples were mounted on a temperature‐controlled Linkam stage. Each sample was heated to 145°C at 20°C min^−1^, equilibrated for 35 min, and then measured at 145°C. The sample was subsequently cooled to 27°C, equilibrated for 10 min, and measured again at 27°C. For in situ tensile SAXS experiments, rectangular bars (20 mm × 2 mm × 1 mm) were mounted on a Linkam Modular Force Stage. Scattering patterns were collected at 0% and 25% engineering strain. Deformation was applied at 40°C at a strain rate of 0.5% s^−1^; at each target strain, samples were held for 1 min prior to data acquisition.

### 4.7 Vat Photopolymerization 3D Printing

A lab‐built high‐resolution single digit micrometer continuous liquid interface production (CLIP) printer was used for all the 3D printing experiments [38]. The setup features a 385 nm ultraviolet projector (3DLP9000, Light Innovations, TX) and a 2X objective lens (Edmund Optics, NJ), providing a projected pixel resolution of 3.5 µm at the projection plane. The build area measures 8.96 mm × 5.6 mm, and a translational stage (Newport, CA) enables a minimum layer thickness of 1 µm. The 3D models were sliced into 1 µm layers using Netfabb. Prior to printing, ∼1 mL of the resin was transferred into the vat, and the stage was lowered to contact the resin, after which the projected sliced patterns were sequentially exposed to fabricate the object layer by layer. A programmed initial exposure defined the first layer, establishing the foundation of the structure. A dark time of 200 ms was incorporated between each exposure to allow resin redistribution and ensure consistent monomer concentration for uniform polymerization [38, 40]. After printing, the object was removed from the build platform and rinsed with toluene to remove residual uncured resin.

### 4.8 Molar Volume Calculation

Molar volume for each polymer was determined by dividing the polymer density by repeat unit molar mass. Polymer densities for PPC and poly(cyclohexene oxide) (PCHC) are 1.26 and 1.10 g cm^−3^, as reported by chemical suppliers (Sigma, Empower Materials). We consider PCHC to have comparable density to PVCHC. Using these values, we calculated molar volumes of 81.0 and 152.8 cm^3^ mol^−1^ for PPC and PVCHC, respectively.

## Author Contributions

**Chia‐Min Hsieh**: writing – original draft, writing – review and editing, visualization, conceptualization, methodology, data curation, investigation. **Krista G. Schoonover**: software, methodology, investigation, data curation, writing – review and editing. **Naushad Ahmed**: methodology. **Jung‐Bin Ahn**: software, data curation, investigation, writing – review and editing. **Shuo Qian**: writing – review and editing, formal analysis, validation. **Michael J. A. Hore**: writing – review and editing, formal analysis, validation. **Weiling Xia**: investigation, methodology, writing – review and editing. **Kaiwen Hsiao**: writing – review and editing, resources, funding acquisition. **Yue Yuan**: funding acquisition, resources. **Mani Sengoden**: methodology. **Donald J. Darensbourg**: conceptualization, writing – review and editing, funding acquisition, supervision, resources. **Emily Pentzer**: conceptualization, writing – review and editing, funding acquisition, supervision, resources. **Peiran Wei**: conceptualization, investigation, writing – original draft, methodology, validation, visualization, writing – review and editing, software, formal analysis, project administration, data curation, supervision, resources.

## Conflicts of Interest

The authors declare no conflicts of interest.

## Supporting information




**Supporting File 1**: anie73315‐sup‐0001‐SuppMat.docx.

**Supporting File 2**: anie73315‐sup‐0002‐VideoS1.mp4.

**Supporting File 3**: anie73315‐sup‐0003‐VideoS2.mp4.

## Data Availability

The data that support the findings of this study are available from the corresponding author upon reasonable request.
