# Supplementary material for: Tuning the Mechanical Properties of Crosslinked Copolymers via Sequence and Solvent‐Selective Swelling for Vat Photopolymerization
Source: Angew Chem Int Ed Engl. 2026 Jun 24;65(35):e8144140. doi: 10.1002/anie.8144140 (PMC13502578; doi:10.1002/anie.8144140)
Supplement: Supplementary file 1 [file ANIE-65-e8144140-s002.docx]

**Supporting Information**

**Tuning the Mechanical Properties of Crosslinked Copolymers via** **Sequence and Solvent-Selective Swelling for Vat Photopolymerization**

**Chia-Min Hsieh,**^†,1^ **Krista G. Schoonover,**^†,1^ **Naushad Ahmed,**^†,1^ **Jungbin Ahn,**^2^ **Shuo Qian,**^3^ **Michael J. A. Hore,**^4^ **Weiling Xia,**^5^ **Kaiwen Hsiao,**^5^ **Yue Yuan,**^6^ **Mani Sengoden,**^1^ **Donald J. Darensbourg,**^1,^**^*^ Emily Pentzer,**^1,7,^**^*^ Peiran Wei**^2,7,^**^*^**

^1^*Department of Chemistry, Texas A&M University, College Station, Texas 77843, USA*

^2^*Soft Matter Facility, Department of Materials Science and Engineering, Texas A&M University, College Station, TX 77843, USA*

^3^*Neutron Scattering Division and Second Target Station, Oak Ridge National Laboratory, Oak Ridge, Tennessee 37831, United States*

^4^*Department of Macromolecular Science and Engineering, Case Western Reserve University, 10900 Euclid Avenue, Cleveland, Ohio 44106, United States*

^5^*Department of Materials Science and Engineering, Texas A&M University, College Station, Texas 77843, USA*

^6^*Center for Nanophase Materials Sciences, Oak Ridge National Laboratory, Oak Ridge, TN 37830, USA*

^7^*Lead contact*

†These authors contributed equally

*Corresponding Authors: [djdarens@chem.tamu.edu](mailto:djdarens@chem.tamu.edu), [emilypentzer@tamu.edu](mailto:emilypentzer@tamu.edu), [peiran@tamu.edu](mailto:peiran@tamu.edu)

Figure S1. SEC trace of PPC.

Figure S2. SEC trace of PVCHC.

Figure S3. SEC trace of ABA.

Figure S4. SEC trace of BAB.

Figure S5. SEC trace of Stat.

Figure S6. Successful formation of an ABA-type triblock copolymer. Bottom: ^1^H NMR spectra in CDCl_3_, with the black trace corresponding to the B block precursor and the red trace showing the ABA triblock copolymer after chain extension. Inset: SEC traces of the B block (black) and the resulting ABA triblock copolymer (red).

Figure S7. Successful formation of a BAB-type triblock copolymer. Bottom: ^1^H NMR spectra in CDCl_3_, with the gray trace corresponding to the A block precursor and the pale red trace showing the BAB triblock copolymer after chain extension. The emergence of new signals associated with the B block confirms successful chain growth. Inset: SEC traces of the A block precursor (black) and the resulting BAB triblock copolymer (pale red).

Figure S8. ^1^H NMR spectrum of PPC in CDCl_3_.

Figure S9. ^1^H NMR spectrum of PVCHC in CDCl_3_.

Figure S10. ^1^H NMR spectrum of ABA in CDCl_3_.

Figure S11. ^1^H NMR spectrum of BAB in CDCl_3_.

Figure S12. ^1^H NMR spectrum of Stat in CDCl_3_.

Figure S13. Offset FTIR spectra of the uncrosslinked and crosslinked copolymers.

Equation S1. Calculation of vinyl content (mmol g^-1^) in copolymers based on the molar ratio of VCHC and PC repeat units.

$$\boldsymbol{vinyl content}\left( \frac{\boldsymbol{mmol}}{\boldsymbol{g}} \right)\boldsymbol{=}\frac{\boldsymbol{1000*VCHC mol\%}}{\boldsymbol{(168*VCHC mol\%+102*PC mol\%)}}$$

Figure S14. TGA/DTG weight loss profiles of the uncrosslinked and crosslinked copolymers.

Figure S15. SLS analysis of PPC in β solvent (propylene carbonate). (A) Angular dependence of Kc/R vs sin^2^(θ/2) at different concentrations. (B) Concentration dependence of Kc/R at a fixed scattering angle. (C) Zimm representation showing combined angular and concentration dependence. (D) Double extrapolation to zero angle and zero concentration to determine *M*_w_, R_g_, and A_2_.

Figure S16. SLS analysis of PVCHC in α solvent (1,2,4-trichlorobenzene). (A) Angular dependence of Kc/R vs sin^2^(θ/2) at different concentrations. (B) Concentration dependence of Kc/R at a fixed scattering angle. (C) Zimm representation showing combined angular and concentration dependence. (D) Double extrapolation to zero angle and zero concentration to determine *M*_w_, R_g_, and A_2_.

Figure S17. SLS analysis of ABA in β solvent (propylene carbonate). (A) Angular dependence of Kc/R vs sin^2^(θ/2) at different concentrations. (B) Concentration dependence of Kc/R at a fixed scattering angle. (C) Zimm representation showing combined angular and concentration dependence. (D) Double extrapolation to zero angle and zero concentration to determine *M*_w_, R_g_, and A_2_.

Figure S18. SLS analysis of ABA in α solvent (1,2,4-trichlorobenzene). (A) Angular dependence of Kc/R vs sin^2^(θ/2) at different concentrations. (B) Concentration dependence of Kc/R at a fixed scattering angle. (C) Zimm representation showing combined angular and concentration dependence. (D) Double extrapolation to zero angle and zero concentration to determine *M*_w_, R_g_, and A_2_.

Figure S19. SLS analysis of BAB in β solvent (propylene carbonate). (A) Angular dependence of Kc/R vs sin^2^(θ/2) at different concentrations. (B) Concentration dependence of Kc/R at a fixed scattering angle. (C) Zimm representation showing combined angular and concentration dependence. (D) Double extrapolation to zero angle and zero concentration to determine *M*_w_, R_g_, and A_2_.

Figure S20. SLS analysis of BAB in α solvent (1,2,4-trichlorobenzene). (A) Angular dependence of Kc/R vs sin^2^(θ/2) at different concentrations. (B) Concentration dependence of Kc/R at a fixed scattering angle. (C) Zimm representation showing combined angular and concentration dependence. (D) Double extrapolation to zero angle and zero concentration to determine *M*_w_, R_g_, and A_2_.

Figure S21. SLS analysis of Stat in β solvent (propylene carbonate). (A) Angular dependence of Kc/R vs sin^2^(θ/2) at different concentrations. (B) Concentration dependence of Kc/R at a fixed scattering angle. (C) Zimm representation showing combined angular and concentration dependence. (D) Double extrapolation to zero angle and zero concentration to determine *M*_w_, R_g_, and A_2_.

Figure S22. SLS analysis of Stat in α solvent (1,2,4-trichlorobenzene). (A) Angular dependence of Kc/R vs sin^2^(θ/2) at different concentrations. (B) Concentration dependence of Kc/R at a fixed scattering angle. (C) Zimm representation showing combined angular and concentration dependence. (D) Double extrapolation to zero angle and zero concentration to determine *M*_w_, R_g_, and A_2_.

Figure S23. Photorheology of ABA, BAB, and Stat resins at 300 mg mL^-1^ in (A-C) solvent β and (D-E) solvent α. Solid and dashed lines denote storage and loss modulus, respectively; gelation times are indicated.

Figure S24. Photograph of BAB (*M*_w,SEC_ ≈ 40 kDa; A:B ≈ 1:1) in β solvent showing fragmented, uneven films.

Figure S25. Variable-temperature SAXS of crosslinked networks. (A, D) xABA; (B, E) xBAB; (C, F) xStat. Top row: solvent β; bottom row: solvent α.

Figure S26. 2D SAXS patterns of (A) xBAB_β_ and (B) xBAB_α_ before and after uniaxial stretching for 5 mm.

Table S1. Mechanical properties of ABA, BAB, and Stat copolymers crosslinked in solvent β, α, and α:β mixed solvent (3:1 by volume), and solvent α.

|  | **Tensile Modulus (MPa)** | **Ultimate Strength (MPa)** | **Stain at**  **Break (%)** | **Toughness**  **(MJ/m^3^)** |
| --- | --- | --- | --- | --- |
| xABA_β_ | 230 ± 30 | 6.7 ± 0.9 | 50 ± 10 | 2.58 ± 0.6 |
| xBAB_β_ | 330 ± 80 | 1.0 ± 0.2 | 0.5 ± 0.3 | 0.003 ± 0.002 |
| xStat_β_ | 56 ± 7 | 11.2 ± 0.9 | 124 ± 1 | 4.9 ± 0.2 |
| xABA_m_ | 230 ± 20 | 10 ± 1 | 65 ± 2 | 4.6 ± 0.5 |
| xBAB_m_^*^ | 100 ± 20 | 4.1 ± 0.2 | 35 ± 2 | 1.2 ± 0.1 |
| xStat_m_ | 49 ± 7 | 15.4 ± 0.6 | 118 ± 3 | 5.7 ± 0.3 |
| xABA_α_ | 140 ± 20 | 19 ± 3 | 110 ± 2 | 9.1 ± 0.7 |
| xBAB_α_^*^ | 90 ± 20 | 7 ± 2 | 60 ± 10 | 2.9 ± 0.8 |
| xStat_α_ | 53 ± 6 | 16 ± 2 | 120 ± 10 | 5.9 ± 0.3 |
| * xBAB_m_ and xBAB_α_ cracked upon drying, leading to significant error expected in mechanical properties | | | | |

Figure S27. Photos of cracked samples.

Figure S28. Summary maps of tensile modulus for ABA, BAB, and Stat cured in solvent β, solvent β:α (1:3), and solvent α.

Figure S29. SEM images of printed structures: (A) gyroid with ABA_α_, (B) bear with ABA_β_, (C) pyramid with Stat_α_, and (D) TAMU logo with Stat_β_.

Table S2. Print parameter for different design geometries in microCLIP printing

| Design | Initial exposure time (s) | Initial exposure intensity (mW/cm^2^) | Layer exposure intensity (mW/cm^2^) | Layer exposure time (ms) |
| --- | --- | --- | --- | --- |
| Octahedron | 26 | 25.1 | 18.9 | 40 |
| Gyroid | 27 | 25.1 | 16.5 | 45 |
| Bear | 30 | 25.1 | 21.4 | 55 |
| Pyramid | 26 | 25.1 | 21.4 | 55 |
| TAMU logo | 30 | 25.1 | 21.4 | 55 |
